# The borderline effect for diabetes: when no difference makes a difference

**DOI:** 10.3389/fpsyg.2024.1333248

**Published:** 2024-05-03

**Authors:** Peter Aungle, Ellen Langer

**Affiliations:** Department of Psychology, Harvard University, Cambridge, MA, United States

**Keywords:** diabetes, diagnostic labels, self-fulfilling beliefs, perceived risk, perceived control

## Abstract

We hypothesized that people at the borderline of being labeled as “prediabetic” based on A1c blood test results, who initially face equivalent risks of developing diabetes but who are labeled differently, would be more likely to develop diabetes when labeled as “prediabetic” as a result of the label. Study 1 served to establish the psychological effect of the prediabetes label: we surveyed 260 participants on Amazon Mechanical Turk to test whether risk perception significantly increased when comparing A1c test results that differed by 0.1% and led to different diagnostic labels (5.6 and 5.7%) but did not significantly increase when comparing those that differed by 0.1% but received the same label (5.5%/5.6 and 5.7%/5.8%). Study 2 explored whether labels are associated with different rates of developing diabetes when the initial difference in A1c results suggests equivalent risk. Using data from 8,096 patients, we compared patients whose initial A1c results differed by 0.1% and found those who received results labeled as prediabetic (A1c of 5.7%) were significantly more likely to develop diabetes than patients whose initial results were labeled as normal (5.6%). In contrast, patients whose initial results differed by 0.1% but who received the same “normal” label (5.5 and 5.6%) were equally likely to develop diabetes. These preliminary results suggest that diagnostic labels may become self-fulfilling, especially when the underlying pathology of patients receiving different labels does not meaningfully differ.

## Introduction

Two marathon runners are nearing the end of a marathon. One notices that they are just over 3 h and 52 min into the race with about a mile to go (a pace of around 9 min/mile). They pick up their pace for the final stretch and finish in just under 4 h. The other runner notices that they are around 3 h and 55 min into the race with three quarters of a mile to go. They quickly calculate they would need to run significantly faster than they had been and feel fatigued by the thought. They slow down and end up finishing in 4 h and 5 min. Pick any marathon, and if one looks at a distribution of finish times, they will invariably find a spike just under 4 h that quickly falls off after the 4-h mark. Being “someone who finishes marathons in under 4 h” apparently motivates runners on the borderline to pick up their pace. But when that positive label is perceived to have fallen out of reach, the extra motivation dissipates and can even reverse (Allen et al., 2017). Such is often the effect of labels: they influence how we make sense of experience and consequently shape behavior, affect, and physiology (Rosenhan, 1973; Chanowitz and Langer, 1981; Crum and Langer, 2007; Langer, 2009; Levy et al., 2009; Crum et al., 2011; Turnwald et al., 2019; Levy, 2022).

In the studies described below, we sought to answer a simple question: given two patients with nearly equivalent results on a diagnostic test, but who differ enough to warrant different diagnostic labels, what, if any, are the effects of the diagnostic label? We tested the effect of diagnostic labels on health trajectories and outcomes by comparing cases in which initial diagnostic labels suggested different risks but the underlying test results did not. Since we cannot randomly assign participants to receive true or false diagnostic test results, the purpose of study 1 was to directly explore the psychology of the borderline effect. To explore the health consequences of this phenomenon, in study 2 we partnered with a university hospital in the Boston metropolitan area to obtain retrospective data from patients who entered the university healthcare system on the border of “normal” and “prediabetic.”

We began our paper with the example of the marathon runners because their behavior nicely captures the interplay of categorical thinking, expectations, and behavior in a way that we think parallels the psychology of “the borderline effect” – many of the runners who finished under 4 h were initially barely ahead of the runners who ended up finishing well over 4 h, but the slightly slower runner’s past experiences, beliefs, and expectations were enough to significantly shift their behavioral calculus (and outcome). The ability of such a small underlying difference in physiology to radically diverge over time speaks to the importance of psychological influences. None of the following is intended as an argument against diagnostic labels or efforts to create early warning systems, but with any continuous variable that has been divided into different categories based on certain thresholds, the borderline between categories becomes increasingly less meaningful the closer the results are to the borderline (Langer, 2023).

We sought to compare cases in which initial diagnostic labels suggested different risks but the underlying test results did not. In the present study we focused on the diagnostic labels used to identify individuals at risk of developing type 2 diabetes. Diabetes diagnoses have almost quadrupled globally over the past three decades, making diabetes one of the most important international public health challenges, affecting more than 460 million people and costing nearly $760 billion globally in 2019 alone (Williams et al., 2020; Ong et al., 2023). Ninety percent of diagnosed diabetes cases are considered type 2, in which the body fails to generate sufficient insulin or fails to use it properly (Xu et al., 2018).

Given the potential short-term and long-term complications that can result from diabetes – including strokes, neuropathies, kidney disease, and vision problems (Deshpande et al., 2008) – it is noteworthy that psychological influences appear to shape the physiology of the illness. For example, stress has been consistently associated with higher blood glucose levels among nondiabetics and diabetics alike (Surwit et al., 1992). Similarly, depression (Van Dooren et al., 2013), and psychological comorbidities more generally (Egede and Dismuke, 2012), negatively affect diabetic physiology. Some of the most interesting evidence that psychological influences alone can shape diabetes-related physiology comes from studies that found blood sugar levels followed perceived time, independent of actual time (Park et al., 2016) and perceived sugar content, independent of actual sugar content (Park et al., 2020). Collectively, these findings provide compelling evidence that psychological factors shape the underlying pathology characteristic of type 2 diabetes.

In this paper, we first studied whether the perception of risk is more sensitive to changes in diagnostic labels than it is to equal underlying changes in hemoglobin A1c (A1c) test results. Given patients cannot be randomly assigned to receive true or false lab test results in real life, we sought to establish whether people only respond differently to small differences in diagnostic test results if those differences also correspond to different diagnostic labels.

## Study 1

We recruited participants to complete a survey on Amazon’s Mechanical Turk and asked participants to imagine receiving two A1c results that differed by the smallest possible value (0.1%). We advertised the study as seeking to better understand how patients process diagnostic test results and recruited adults in the United States between the ages of 30 and 65. Participants completed a survey that asked how they imagined they would feel and act after receiving two nearly equivalent diagnostic test results. We received 322 completed surveys and eliminated those that failed an attention check or spent fewer than 20 s completing the survey (Goodman et al., 2013), resulting in a final sample of 260 participants (175 male, average age = 40.8 years [SD = 7.54]). Previous research has found that mTurk provides greater demographic diversity relative to alternative recruitment methods (Berinsky et al., 2012), and our power analysis for a mixed between within-subject design with three groups, two repeated measures, and an assumed medium effect size indicated approximately 80 participants per group would provide sufficient power to detect an effect (Zhang et al., 2018). Participants who responded to the advertisement and met our eligibility criteria were directed to a survey that first collected informed consent, after which it randomly assigned them to one of three conditions: one in which they imagined A1c test results that both corresponded to “normal” labels, one in which they imagined one “normal” result and one “prediabetic” result, or one in which they imagined A1c results that both corresponded to “prediabetic” labels. Within each condition, A1c test results were counterbalanced – half the participants saw the higher of the two results first, the other half saw the lower of the two results first. In the first group, participants imagined receiving results of either 5.5% or 5.6% (both labeled as “normal”). In the second group, they imagined receiving results of either 5.6% or 5.7% (the first was labeled as “normal,” the second was labeled as “prediabetic”). In the third group, they imagined receiving results of either 5.7% or 5.8% (both labeled as “prediabetic”). For each A1c test result that participants imagined receiving, they responded to four survey items that asked them about their perceived likelihood of developing diabetes, the degree of worry they would feel, the agency they’d feel to take effective preventative action, and the efficacy they perceived in preventative medical care.

We predicted that the only significant within-group differences would occur in the second condition, in which the 0.1% difference corresponded to different diagnostic labels: “normal” vs. “prediabetic.”

### Measures

#### Risk

Assessed impact of the two test results on perceived risk of developing diabetes. “Compared to most people your age and sex, what would you say your chances are for developing diabetes?” (1 = very unlikely, 6 = very likely).

#### Worry

Asked participants to rate their concern about developing diabetes. “I would be worried about developing diabetes” (1 = strongly disagree, 6 = strongly agree).

#### Agency

Assessed the degree to which participants imagined feeling control over the likelihood they would develop diabetes. “There’s a lot I can do to prevent the development of diabetes” (1 = strongly disagree, 6 = strongly agree).

#### Medical care

Assessed the extent to which participants said they would believe that regular medical care would protect them from developing diabetes (1 = strongly disagree, 6 = strongly agree).

### Results

Within-subjects linear models were constructed to test whether responses to our measures were significantly influenced by the A1c test results participants imagined receiving. The measures described above were the outcome variables, A1c score was a categorical predictor, and correlations between repeated measures were accounted for by including a random intercept in each model.

#### Group 1

Participants imagined receiving two A1c test results, both of which corresponded to “normal” labels (5.5 and 5.6%). Half of the participants imagined the higher number first (counterbalanced randomly across participants). We found no differences on any of our four measures.

**Table 1:** 

Group 1 | Normal Labels | *N* = 84				
	5.5% (“Normal” A1c)		5.6% (Normal A1c)	
	Mean	SD	Mean	SD
Risk	3.38	1.405	3.40	1.354
Worry	3.70	1.421	3.76	1.453
Agency	4.74	0.983	4.77	1.079
Medical care	4.45	1.186	4.50	1.197

* *p* < 0.05, ** *p* < 0.01, *** *p* < 0.001.

#### Group 2

Participants imagined two A1c test results, one of which corresponded to a “normal” label (5.6%) and the other of which corresponded to a “prediabetic” label (5.7%). Half of the participants imagined the higher number first (counterbalanced randomly across participants). Participants perceived significantly greater risk of developing diabetes (mean difference = 0.556, *t* (89) = 4.87, p < 0.0001) and said they would worry significantly more (mean difference = 0.689, *t* (89) = 5.15, *p* < 0.0001) when they imagined receiving a result of 5.7% (“prediabetic”) compared to when they imagined receiving a result of 5.6%. Results on our measures of perceived agency and the protective value of regular medical care did not significantly differ. In short, participants said they would be more worried but would not behave differently.

**Table 2:** 

Group 2 | Label Change | *N* = 90				
	5.6% (“Normal” A1c)		5.7% (“Prediabetic” A1c)	
	Mean	SD	Mean	SD
Risk***	3.50	1.211	4.06	1.115
Worry***	3.69	1.196	4.38	1.250
Agency	4.67	0.983	4.66	1.018
Medical care	4.60	1.099	4.69	0.979

* *p* < 0.05, ** *p* < 0.01, *** *p* < 0.001.

#### Group 3

Participants imagined two A1c test results, both of which corresponded to “prediabetic” labels (5.7 and 5.8%). Half of the participants imagined the higher number first (counterbalanced randomly across participants). In contrast to our hypothesis, participants perceived greater risk of developing diabetes (mean difference = 0.233, *t* (85) = 2.58, *p* = 0.0116) and said they would worry more (mean difference = 0.267, *t* (85) = 2.51, *p* = 0.0139) when they imagined receiving a result of 5.8% (“prediabetic”) compared to when they imagined receiving a result of 5.7% (“prediabetic”). The more threatening label apparently increased psychological sensitivity to small differences in A1c results, but again participants did not imagine they would behave any differently.

**Table 3:** 

Group 3 | Prediabetic Labels | *N* = 86				
	5.7% (“Prediabetic” A1c)		5.8% (“Prediabetic” A1c)	
	Mean	SD	Mean	SD
Risk*	4.01	1.232	4.24	1.04
Worry*	4.30	1.284	4.57	1.342
Agency	4.58	0.988	4.64	1.084
Medical care	4.52	1.003	4.49	1.155

* *p* < 0.05, ** *p* < 0.01, *** *p* < 0.001.

#### Differences between groups

Applying a mixed between-within-subjects linear model to test for differences between groups, the pattern of results suggests participant perceptions were dominated by the diagnostic label. Similar to the within-group differences we found in Groups 1 and 3, the model indicated a significant between-group effect on perceived risk and worry but not on agency or medical care. Pairwise contrasts with p-values adjusted using the Tukey method indicated that this effect was only significant when comparing the responses from Group 1 to those from Group 3. Participants who responded to results both labeled as “normal” perceived significantly less risk (mean difference = −0.735, *t* (257) = −4.189, *p* = 0.0001) and imagined feeling significantly less worried about developing diabetes (mean difference = −0.704, *t* (257) = −3.778, *p* = 0.0006) than did participants who responded to results both labeled as “prediabetic.”

### Discussion

The purpose of this study was to test whether small differences in A1c test results only result in significantly different responses when they correspond to different diagnostic labels. If participants treated small differences in underlying A1c results equivalently, differences in perceived risk and imagined concern about developing diabetes should have been similar within each group. Comparing perceived risk from a patient with an initial A1c of 5.5% to one with an initial A1c of 5.6% should result in a similar difference as comparing perceived risk from a patient with an initial A1c of 5.7% to one with an initial A1c of 5.6%., but that is not how participants behaved. Participants in Group 1 responded as if the 0.1% difference represented an equivalent result, participants in Group 2 perceived significantly greater risk and imagined worrying significantly more, and participants in Group 3 perceived slightly higher risk and imagined worrying slightly more – the only group that appeared to respond more to the specific A1c result than to the diagnostic label. Thus, the psychological effect of the same 0.1% difference was far from equal.

## Study 2

In Study 1, we established that small differences in A1c results loom disproportionately large when those differences correspond to different diagnostic labels. When both results were labeled normal, the difference in the underlying result was irrelevant. When the label changed, participants perceived significantly greater risk of developing diabetes and said they would worry significantly more. When both results were labeled as prediabetic, participants perceived slightly greater risk and said they would worry slightly more if they received the higher of the two A1c results. This suggests that the “normal” label dominated judgments of A1c results in the first group; that participants in the second group were especially sensitive to small differences because they corresponded to different diagnostic labels; and that participants only began to perceive A1c results as a continuous measure of risk because both results were labeled as “prediabetic” in the third group.

In the retrospective analysis we conducted for Study 2, we tested whether the frequency with which people developed diabetes in a real-life patient population differed based on the label assigned to their initial A1c results. We partnered with the endocrinology team at Tufts Medical Center to develop the study design and to obtain retrospective data for patients whose initial lab results when they entered the system were between 5.5 and 5.8%. Our hypothesis was that A1c trajectories and the frequency with which patients developed diabetes would be significantly worse when the initial A1c results were labeled as “prediabetic” compared to when they were labeled as “normal.”

### Methods

We received data from Tufts Medical Center containing: 32,957 A1c test results from 8,096 patients (3,370 men) who received initial results after the “prediabetes” label was adopted. At the time the data were extracted, the patients were 59 years old on average (SD = 11.83, IQR = 17 years). We grouped patients by initial A1c results and conducted chi-square tests within each group to compare the number of patients who developed diabetes to the number who did not.

Extensive research supports using hemoglobin A1c (HbA1c or A1c) for diagnosing prediabetes and diabetes (World Health Organization, 2011), highlighting its effectiveness in capturing chronic hyperglycemia over about two to three months. The advantages of A1c include its ability to provide a stable indicator of glycemic control, which is less susceptible to daily fluctuations caused by stress, illness, or dietary intake compared to fasting plasma glucose (FPG) levels or an oral glucose tolerance test (OGTT). We chose to use retrospective A1c test result data in our analyses in order to include a large number of patients whose changes in A1c scores were measured multiple times.

### Results

We first limited our analysis according to the design we developed in consultation with the endocrinologists who provided these data: we compared the number of “high normal” patients (initial A1c results of 5.5 and 5.6%) who developed diabetes to the number of “low prediabetic” patients (initial A1c results of 5.7 and 5.8%) who developed diabetes. A chi-square test indicated a significant difference: 109 out of 4,079 patients in the “normal” group developed diabetes compared to 179 out of 3,680 patients in the “prediabetic” group ( $\chi^{2}\left(df=1\right)=$ 24.12, *p* < 0.00001). We then looked at differences in outcomes by grouping patients whose initial A1c results only differed by 0.1%, analogous to the survey design we used in Study 1.

#### Group 1

Compared patients with initial A1c results of 5.5% to patients with initial A1c results of 5.6%. Paralleling the survey results from Study 1, which found no differences in evaluations of 5.5% *vs* 5.6%, the number of patients who developed diabetes was roughly equivalent: 50 out of 2,037 compared to 59 out of 2,042 ( $\chi^{2}\left(df=1\right)=$ 0.702, *p* = 0.402).

#### Group 2

Compared the number of patients with initial A1c results of 5.6% (“normal”) who developed diabetes to the number of patients with initial A1c results of 5.7% who developed diabetes (“prediabetic”). Like the survey study results from Study 1, which found perceived risk and anticipated anxiety significantly increased when the 0.1% difference corresponded to a label change, a chi-square test indicated a significant association between the number of patients who developed diabetes and the label given to their initial A1c results: 59 out of 2,042 compared to 80 out of 1,942 ( $\chi^{2}\left(df=1\right)=$ 4.171, *p* = 0.0411).

#### Group 3

Compared the number of patients with initial A1c results of 5.7% (“prediabetic”) who developed diabetes to the number of patients with initial A1c results of 5.8% (“prediabetic”) who developed diabetes. A chi-square test indicated a significant association between the number of patients who developed diabetes and their initial A1c results: 80 out of 1,942 compared to 99 out of 1,738 ( $\chi^{2}\left(df=1\right)=$ 7.597, *p* = 0.0460). This result is consistent with the results from Study 1, which found that perceived risk and anticipated concern were more sensitive to different A1c results when the results were no longer labeled “normal.”

One possible explanation for these results is that A1c values above 5.6% accurately reflect a critical level above which patients are at significantly higher risk of developing diabetes – i.e., that the observed associations are due to the differences in A1c results, not differences in diagnostic labels. If that were the case, we would expect the same pattern of results when comparing patients who received their initial results before the prediabetes label was introduced in 2003. The data we received from Tufts Medical Center included 1,018 A1c test results from 466 patients that were collected before 2003. When we analyzed these data, none of the comparisons between patients whose initial A1c results differed by 0.1% were significant (all *p*-values >0.14), suggesting that the psychological differences highlighted by Study 1 are more than mere coincidence: Study 2 patients whose A1c results initially bordered on “normal” seemed to experience significantly different outcomes depending on whether they were labeled as “normal” or as “prediabetic.” Whether patients initially on the borderline of “normal” in other disease contexts similarly experience significantly different trajectories is an interesting empirical question that warrants further research.

### General discussion

The Pygmalion Effect, as explored by Rosenthal and Jacobson (1968), provides a compelling framework for understanding how expectations and labels can shape behavior and outcomes. When teachers were led to believe certain students were destined to excel (labeled as “bloomers”), these students performed significantly better academically, influenced by the teachers’ heightened expectations and, likely, the differential treatment that followed and underscoring the power of labels to not only reflect but also dictate reality through a self-fulfilling prophecy (Rosenthal and Jacobson, 1968). Translating this effect to the medical realm, particularly in the context of “prediabetes,” we can see how diagnostic labels might similarly influence patient and healthcare provider behaviors. Being labeled as “normal” could instill a sense of self-efficacy and calm, allowing patients who might be motivated to maintain their “normal” status to respond more adaptively to their test results, and it might unwittingly influence clinicians’ expectations and behavioral interactions.

Conversely, negative labels are known to have damaging effects. For example, describing problematic use of drugs and alcohol as “Substance Use Disorder” rather than “Substance Abuse Disorder” significantly affects perceived blame and willingness to treat people struggling to change harmful habits. Research has highlighted how this change in terminology (Hasin et al., 2013) helps reduce stigma and blame, facilitating a more supportive environment for individuals seeking help (Kelly and Westerhoff, 2010; Botticelli and Koh, 2016).

Taken together, the results from Studies 1 and 2 suggest the similarly powerful effects labels have on interpretations of A1c test results. None of the foregoing is intended as an argument against diagnostic labels *per se*. Rather, it is intended to highlight the importance of considering how such labels are applied, the ambiguity labels tend to hide, and the effects they have on both patients and clinicians alike. Simple changes in semantic connotations can have profound effects on psychology and behavior. Consider a few examples. Patients receiving emergency care just after their 40^th^ birthday compared to just before are 10% more likely to be screened for and 20% more likely to be diagnosed with Ischemic Heart Disease, reducing the number of missed diagnoses and increasing the probability of receiving lifesaving medical care (Coussens, 2018). Patients who need coronary-artery bypass grafting (CABG) surgery are significantly more likely to receive it if they happen to see their doctor 2 weeks before their 80^th^ birthdays compared to 2 weeks after (Olenski et al., 2020). Like the patients with A1c results just at the borderline of indicating “prediabetes,” these examples illustrate the consequences of thinking categorically and the importance of the language used to distinguish between categories. If the purpose of the “prediabetes” label is to encourage lifestyle changes known to mitigate cardiovascular and diabetic health risks (Diabetes Prevention Program (DPP) Research Group, 2002) – risks similarly faced by patients whose A1c results are labeled “normal” but who border on “prediabetes” – this study suggests we may need a more nuanced vocabulary to interpret A1c test results.

## Conclusion

The ability to categorize is fundamental to human intelligence and adaptive functioning (Ryan, 1995; Goldstone and Hendrickson, 2010). Diagnostic labels facilitate effective medical care (Engel, 1977; Blaxter, 1978; Jutel, 2009), but like any form of categorization, they tend to obscure the blurriness between categories (Mervis and Rosch, 1981). Whether the consequences of the borderline effect in diabetes manifest because patients make distorted inferences (Sims et al., 2021), doctors use oversimplified heuristics (Coussens, 2018; Olenski et al., 2020), or some combination thereof is an interesting empirical questions in need of future research. Additionally, we hope other researchers will explore the borderline effect in other health contexts such as high blood pressure, high cholesterol, and so on. These data suggest no difference can make a difference when it leads to categorical thinking that inhibits the ability to appreciate the blurry boundaries between categories.

## Data availability statement

The datasets presented in this article are not readily available because we do not currently have permission to share the retrospective patient data from Tufts Medical Center. We are happy to share the data from Study 1 and can seek approval to share the retrospective data with approved third parties. Requests to access the datasets should be directed to peter_aungle@fas.harvard.edu.

## Ethics statement

The studies involving humans were approved by Harvard University Institutional Review Board Committee on the Use of Human Subjects. The studies were conducted in accordance with the local legislation and institutional requirements. The participants provided their written informed consent to participate in this study.

## Author contributions

PA: Writing – review & editing, Writing – original draft, Methodology, Investigation, Formal analysis, Conceptualization. EL: Writing – review & editing, Conceptualization.
